# A comprehensive pilot care program for people suffering with chronic pain, opioid analgesic control problems and other mental disorders

**DOI:** 10.1192/j.eurpsy.2025.316

**Published:** 2025-08-26

**Authors:** T. Bollain Muñoz, R. Crespo Rubio, L. Gonzalez Hernandez, M. L. Barrigón Estevez, J. Guerra del Peso, N. Szerman, M. D. L. M. Peña Granger, A. Gimenez Manzorro, L. Lopez Fernandez

**Affiliations:** 1 Psychiatry, HGUGM, Madrid, Spain

## Abstract

**Introduction:**

Some individuals with chronic pain using opioid analgesics may unknowingly experience mood improvement, which can lead to compulsive opioid use. To address this issue, the DOLMEN program was developed, involving a multidisciplinary team of clinical psychologists, psychiatrists, pain specialists, nurses, pharmacists, and geneticists for a comprehensive treatment approach.

**Objectives:**

Our objective was to describe the DOLMEN program and assess its effectiveness in improving emotional pain, psychiatric symptoms, functioning, quality of life, and pain management. The program also aimed to prevent medication errors through pharmacological guidance. We investigated genetic biomarkers related to clinical outcomes and evaluated patient satisfaction with the program.

**Methods:**

We implemented a multidisciplinary pilot program and conducted an observational, unicentric, longitudinal, and ambispective study to evaluate its outcomes. The study included patients with chronic pain on high off-label opioid doses, those with mental disorders treated with opioids, and patients with pain refractory to standard treatments. Data were collected on psychiatric symptoms, substance use, CYP2D6, CYP2C19, and OPRM1 gene polymorphisms, and pharmacist interventions. To assess the intervention’s impact, a paired test analyzed changes in psychiatric measures and pain levels using the Visual Analog Scale (VAS) after structured psychoeducational sessions. A linear-by-linear association chi-square test examined associations between genetic polymorphisms and questionnaire results.

**Results:**

The sample consisted of 90 patients, the majority of whom were women (78.0%), with a mean age of 53.1 years. Among the participants, 23.0% exhibited problematic opioid use.

Significant improvements were observed in several clinical measures. The average Clinical Global Impression score improved from 3.7 to 2.58 (p=0.003), while the State-Trait Anxiety Inventory scores for state anxiety and trait anxiety decreased from 38.8 to 34.9 (p=0.0027) and from 40.1 to 35.8 (p<0.0001), respectively. The Beck Depression Inventory-II scores also showed a notable reduction from 33.1 to 27.8 (p<0.0001). Although improvements were seen in the EQ-5D-5L (from 41.1 to 50.2) and the EEAG (from 65.8 to 71.8), these changes were not statistically significant (p>0.05). Pain experience, as measured by the Visual Analog Scale, showed a reduction from 7.1 to 6.6 (p=0.0106). An association was identified between the OPRM1 polymorphism and the degree of problematic opioid use, as well as between the CYP2C19 genotype and the presence of depression (p<0.05). Additionally, pharmacists provided 64 recommendations to patients to prevent medication errors throughout the program.

**Conclusions:**

The DOLMEN program demonstrated preliminary effectiveness in managing patients with chronic and emotional pain, as well as problematic opioid use.

**Disclosure of Interest:**

None Declared

